# Clinical Outcomes of Oral Anticoagulation in Elderly East Asian Patients with Atrial Fibrillation: A Retrospective Single-Center Study

**DOI:** 10.3390/life15081298

**Published:** 2025-08-15

**Authors:** Kyunyeon Kim, YouMi Hwang, Sang-Suk Choi, Hunjoo Lee, Young-Jun Son, Myungjae Yoo

**Affiliations:** 1 Department of Cardiology, St. Vincent’s Hospital, The Catholic University of Korea, Seoul 16247, Republic of Korea; gemini729@naver.com (K.K.); sader500@naver.com (S.-S.C.); 8777667@hanmail.net (H.L.); son8790@naver.com (Y.-J.S.); sweetmilkiss@naver.com (M.Y.); 2 Catholic Research Institute for Intractable Cardiovascular Disease (CRID), College of Medicine, The Catholic University of Korea, Seoul 06591, Republic of Korea

**Keywords:** atrial fibrillation, oral anticoagulants, elderly patients

## Abstract

(1) Background: Atrial fibrillation (AF) is the most common arrhythmia and poses a clinical dilemma in the very elderly due to increased thromboembolic and bleeding risks. This study aimed to evaluate clinical outcomes—including thromboembolic events, major bleeding, and all-cause mortality—by age group in elderly East Asian patients with non-valvular AF receiving oral anticoagulants. (2) Methods: This retrospective single-center study included 502 patients aged ≥70 years treated with direct oral anticoagulants (DOACs: dabigatran, rivaroxaban, edoxaban, or apixaban) or warfarin between 2016 and 2024. Patients were stratified into two age groups: 70–79 and ≥80 years. The primary outcomes were ischemic stroke, systemic thromboembolism, and major bleeding. (3) Results: Although patients aged ≥80 years showed a numerically higher incidence of bleeding in both the DOAC and warfarin groups, these differences were not statistically significant after multivariable adjustment (DOAC group: HR 0.832; 95% CI, 0.456–1.518; *p* = 0.549; warfarin group: HR 3.617; 95% CI, 0.600–21.804; *p* = 0.161). Ischemic and thromboembolic event rates were also comparable between age groups. (4) Conclusions: Despite a numerically higher bleeding risk in the very elderly, DOACs remained safe and effective when appropriately managed. These findings support individualized anticoagulation decisions based on clinical factors rather than age alone in elderly East Asian patients with AF.

## 1. Introduction

Atrial fibrillation (AF) is the most common cardiac arrhythmia, showing steadily increasing global prevalence, particularly among older adults [1]. AF significantly impairs quality of life and contributes to substantial healthcare expenditures, posing a considerable socioeconomic burden [2]. Compared to the general population, patients with AF have an approximately fivefold increased risk of ischemic stroke [1]. Advanced age is one of the strongest risk factors of ischemic stroke in patients with AF, making oral anticoagulation a cornerstone of stroke prevention in older adults [3]. This elevated risk is partly attributable to age-related atrial remodeling—including fibrosis, myocyte hypertrophy, and electrical changes—which promotes a prothrombotic and arrhythmogenic substrate in elderly patients with AF [4]. However, anticoagulant therapy in the elderly presents a unique clinical challenge. Due to comorbidities such as chronic kidney disease, liver diseases, concomitant use of antiplatelet agents, or a history of bleeding, these patients often have an increased risk of bleeding [5,6,7]. Clinicians may therefore hesitate to prescribe anticoagulation, leading to potential undertreatment and suboptimal outcomes in this high-risk population [8].

The introduction of direct oral anticoagulants (DOACs) has made available a safer and more convenient alternative to warfarin [9,10,11,12]. Although randomized controlled trials have demonstrated the efficacy and safety of DOACs in the general AF population, data specifically focusing on very elderly patients remain limited [13]. Real-world evidence assessing the risk of thromboembolic and bleeding events in patients aged ≥80 years receiving DOACs or warfarin is still insufficient [14].

Moreover, during antithrombotic therapy, East Asian patients have a distinct risk–benefit profile, with higher bleeding susceptibility and lower anti-ischemic efficacy [15,16]. However, whether these findings extend to very elderly East Asian patients remains unclear, highlighting the need for population-specific studies [17]. In particular, patients aged 80 years or older remain markedly underrepresented in major randomized trials and observational registries, despite their high prevalence of AF and elevated bleeding risk. Addressing this evidence gap is essential for guiding real-world anticoagulation strategies in this vulnerable population [9,10,11,12,18,19,20,21,22,23]. Therefore, in this study, we aimed to evaluate clinical outcomes—including ischemic stroke, thromboembolic events, bleeding complications, and all-cause mortality—among patients aged ≥70 years with non-valvular AF, stratified by age group (70–79 vs. ≥80 years), in order to assess the impact of advanced age on the safety and effectiveness of oral anticoagulation therapy in elderly East Asian patients.

## 2. Methods

### 2.1. Study Design and Population

**Figure 1:**
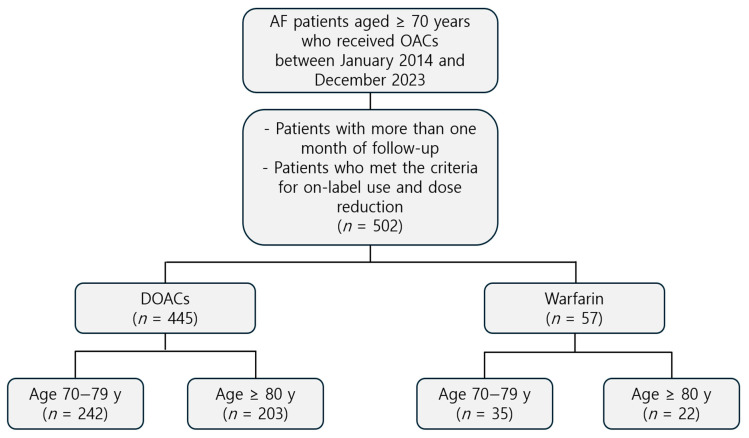
Study population flow chart.

This retrospective, single-center study was conducted at St. Vincent’s Hospital, The Catholic University of Korea. All patients aged 70 years or older with a diagnosis of non-valvular atrial fibrillation who were prescribed oral anticoagulants between 1 January 2014 and 31 December 2023 were included in this study. Only patients who had not previously received oral anticoagulation therapy were eligible for inclusion. Eligible patients received anticoagulation therapy with either DOACs (including dabigatran, rivaroxaban, edoxaban, or apixaban) or warfarin during the study period. Those with less than one month of follow-up and patients receiving off-label DOAC doses were excluded from the analysis. A total of 7 patients were lost to follow-up within the first month and were excluded from the final analysis, leaving 501 patients for the outcome assessment. Only patients who received DOACs according to on-label indications and met established criteria for dose reduction, based on current guideline recommendations, were included in the study [1]. Data on demographic characteristics, baseline comorbidities, and cardiovascular history were collected. Clinical events occurring after the initiation of anticoagulation therapy were recorded. Additional information, including diagnostic imaging, laboratory test results, and concomitant medication use, was also reviewed. Ethical approval was obtained from the Institutional Review Board of St. Vincent’s Hospital, while the study was conducted in accordance with the Declaration of Helsinki (Approval No. VS24RISI0060, approved 21 March 2024). Figure 1 shows the study design flowchart.

### 2.2. Data Collection

Clinical data were retrospectively retrieved from the electronic medical records system of St. Vincent’s Hospital, The Catholic University of Korea. Baseline characteristics collected at the time of anticoagulant initiation included demographic variables (age, sex, height, weight, body mass index, etc.), medical and cardiovascular history (hypertension, diabetes mellitus (DM), congestive heart failure (CHF), prior myocardial infarction (MI), stroke, vascular disease, etc.), history of surgical or interventional procedures, medication use, smoking status, and alcohol consumption. Routine laboratory parameters obtained through standard clinical assessments were also recorded. These included hemoglobin, platelet count, blood urea nitrogen (BUN), serum creatinine, aspartate aminotransferase (AST), alanine aminotransferase (ALT), and B-type natriuretic peptide (BNP) levels. Follow-up data encompassed cardiovascular and cerebrovascular events (e.g., ischemic stroke and myocardial infarction), bleeding complications (including those requiring surgical or procedural intervention), newly prescribed medications, all-cause mortality, and incident disability. All clinical outcomes were identified and confirmed through the chart reviews and discharge summaries, as applicable. In addition, the CHA_2_DS_2_-VA scores were calculated at baseline to assess thromboembolic risk, in accordance with current guidelines for patients with AF [1].

### 2.3. Clinical Outcomes and Definitions

The primary outcomes of this study were the occurrence of ischemic stroke, systemic thromboembolic events, and major bleeding events during the follow-up period. Ischemic stroke was defined as a sudden onset of focal neurological deficit lasting more than 24 h, confirmed by neuroimaging. Systemic thromboembolic events included acute arterial occlusions in non-cerebral sites (e.g., limbs, kidneys, or mesenteric arteries) presumed to be embolic in origin. Major bleeding events were defined per the ISTH bleeding criteria [24].

The secondary outcomes included all-cause mortality and intracranial hemorrhage (ICH). ICH was defined as any spontaneous bleeding within the cranial vault, confirmed by brain imaging, regardless of the presence of clinical symptoms. All-cause mortality encompassed any death from cardiovascular or non-cardiovascular causes that occurred during the follow-up period. All clinical outcomes were monitored throughout the observational period via routine clinical follow-up and thorough review of inpatient and outpatient medical records. Events were identified and validated based on documentation by treating physicians, discharge summaries, and diagnostic imaging or laboratory data, where applicable, and were not formally adjudicated but retrospectively extracted from medical records.

### 2.4. Statistical Analysis

Continuous variables were expressed as the mean with standard deviation (SD) or the median with interquartile range (IQR), as appropriate, based on distributional characteristics. Continuous variables for groups were compared using the Student’s *t*-test for normally distributed data or the Wilcoxon rank-sum test for non-normally distributed data. Categorical variables were presented as frequencies and percentages and compared using the chi-square test or Fisher’s exact test, as applicable. Survival analyses were performed using the Kaplan–Meier method to estimate event-free survival, while comparisons between groups were conducted using the log-rank test. To identify independent predictors of clinical outcomes, multivariable analyses were performed using Cox proportional hazards regression models. Covariates included in the models were selected based on clinical relevance, encompassing demographic, clinical, and procedural characteristics. The proportional hazards assumption was verified using log-minus-log (LML) survival plots and examination of Schoenfeld residuals. Multivariable regression analyses were applied to both primary and secondary outcomes to identify independent prognostic factors. All statistical analyses were performed using SPSS software (version 29.0, IBM Corp., Armonk, NY, USA). A two-sided *p*-value of <0.05 was considered statistically significant.

## 3. Results

### 3.1. Baseline Characteristics

Patients were stratified into two age groups: 70–79 and 80 or older years. A total of 502 patients were included in the analysis, of whom 445 (88.6%) received direct oral anticoagulants (DOACs), while 57 (11.4%) received warfarin. Among DOAC users, 242 (54.4%) were aged 70–79 years, while 203 (45.6%) were aged 80 years or older. Among warfarin users, 35 (61.4%) were aged 70–79 years and 22 (38.6%) were aged 80 years or older. The mean follow-up duration was 2006.9 ± 1081.6 days in the DOAC group and 2454.9 ± 1120.2 days in the warfarin group. Table 1 summarizes the baseline characteristics of the study population. In the DOAC group, patients aged 80 years or older showed significantly lower BMI, height, weight, hemoglobin levels, and estimated glomerular filtration rate (eGFR) compared with those aged 70–79 years (all *p* < 0.05). Similar differences in hemoglobin and eGFR were observed between the age subgroups in the warfarin group. In terms of medical history, the prevalence of smoking was significantly lower in the older age group (*p* = 0.010 for DOAC users). There were no statistically significant differences between the age groups in the prevalence of hypertension, DM, cerebrovascular accident (CVA), CHF, chronic kidney disease (CKD), chronic obstructive pulmonary disease (COPD), or coronary artery disease (CAD) in either treatment group. The CHA_2_DS_2_-VA score was significantly higher in patients aged ≥80 years than those aged 70–79 years in the DOAC group (*p* = 0.003). Within the DOAC group, concomitant antiplatelet use was significantly more frequent in patients aged 70–79 years (*p* = 0.033).

### 3.2. Clinical Outcomes

**Figure 2:**
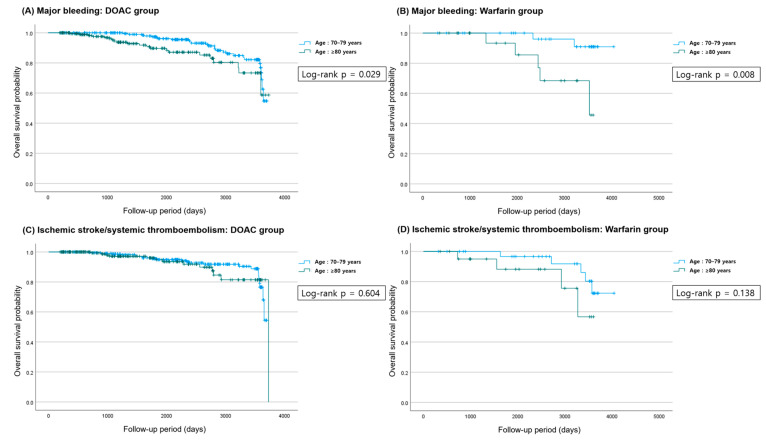

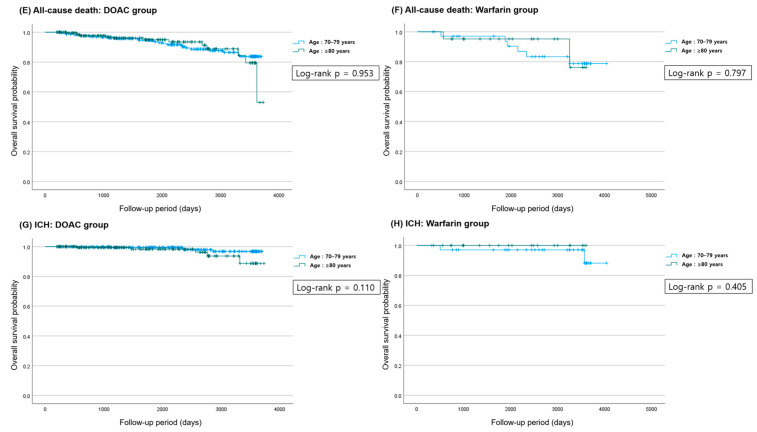
The Kaplan–Meier curves for clinical outcomes during the follow-up periods. Differences between groups were assessed using the log-rank test. Time is shown in days from the initiation of anticoagulation. (**A**) Major bleeding in DOAC group. (**B**) Major bleeding in warfarin group. (**C**) Ischemic stroke or systemic thromboembolism in DOAC group. (**D**) Ischemic stroke or systemic thromboembolism in warfarin group. (**E**) All-cause death in DOAC group. (**F**) All-cause death in warfarin group. (**G**) ICH in DOAC group. (**H**) ICH in warfarin group.

Figure 2 shows the Kaplan–Meier curves for clinical outcomes during the follow-up period. A statistically significant difference in major bleeding was observed between the age groups in both the DOAC group (log-rank *p* = 0.029) and the warfarin group (log-rank *p* = 0.008). In the Kaplan–Meier curves, divergence in major bleeding events between the age groups became apparent in the DOAC group after approximately 1500 days, and in the warfarin group, after 1000 days. For all other outcomes—ischemic stroke/systemic thromboembolism, all-cause death, and intracranial hemorrhage (ICH)—no significant differences were found between the two age groups in either treatment group. Table 2 summarizes the clinical outcomes among patients receiving OACs by age group. Univariate analysis revealed that total bleeding in the DOAC group was associated with a hazard ratio (HR) of 1.885 (95% CI: 1.058–3.360, *p* = 0.031). In contrast, multivariate analysis yielded an adjusted HR of 0.832 (95% CI: 0.456–1.518, *p* = 0.549). Other outcomes in the DOAC group did not show statistically significant differences in either univariate or multivariate analyses. In univariate analysis, major bleeding in the warfarin group was associated with a HR of 3.619 (95% CI: 1.328–16.159, *p* = 0.022), but in multivariate analysis, this was not statistically significant (adjusted HR: 3.617, 95% CI: 0.600–21.804, *p* = 0.161). No other endpoints showed substantial differences between the two age groups in either the unadjusted or adjusted models. Table 3 presents sex differences in clinical outcomes within the DOAC and warfarin groups. No significant differences were observed between men and women in either group.

## 4. Discussion

In this retrospective analysis of elderly patients with atrial fibrillation, we found that clinical outcomes such as all-cause death and ischemic stroke/systemic thromboembolism were not significantly different between patients aged 70–79 years and those aged 80 years or older. However, in the unadjusted analysis, the incidence of major bleeding in patients aged 80 years or older tended to be higher, though after multivariable adjustment, this association was not statistically significant. These results are consistent with previous real-world studies and subgroup analyses from major DOAC trials, supporting the effectiveness and relative safety of DOACs, even in very elderly Asian patients [9,10,11,12,18,19,20,21,22,23].

The efficacy and safety of DOACs in Asian patients with AF have been consistently demonstrated in both randomized controlled trials and real-world data [18,19,20,21,22,23]. Subgroup analyses from major trials, such as RE-LY, ROCKET-AF, ENGAGE AF-TIMI 48, and ARISTOTLE, showed that NOACs significantly reduced the risk of stroke or systemic embolism. In East Asian populations, they were associated with a substantially lower risk of intracranial hemorrhage compared to warfarin [18,20,22,23]. For example, in the RE-LY trial, dabigatran administered at 110 and 150 mg showed a 43–80% reduction in ICH among Asian participants. In contrast, in the ENGAGE AF-TIMI 48 trial, edoxaban (both 30 and 60 mg) was associated with significantly lower rates of major bleeding and stroke in East Asians compared with warfarin [20,22]. Similarly, in the ARISTOTLE trial, apixaban demonstrated a 47% reduction in major bleeding among East Asian patients [18].

These data are further corroborated by observational studies from Korea and other Asian countries, where DOAC use has been associated with lower rates of ICH and all-cause mortality, particularly in elderly populations [25,26,27]. In a Korean national cohort, DOACs showed similar efficacy in preventing ischemic stroke but significantly reduced risks of ICH and death in patients aged ≥75 years [21]. Together, these findings reinforce the notion that DOACs represent a preferable anticoagulation strategy in Asian patients with AF—including the very elderly—and this notion is in agreement with the results of our study.

Although patients aged ≥80 years exhibited numerically higher rates of major bleeding and ICH, these differences were not statistically significant, especially after multivariable adjustment. Although after adjustment, the increased bleeding risk in patients aged 80 years or older was not statistically significant, the observed trend toward higher bleeding rates warrants close clinical attention. With careful patient selection and appropriate dose adjustment, DOACs appear to remain a safe option in elderly patients. However, proactive monitoring is essential, especially for the very elderly. Furthermore, the absence of significant differences in ischemic events in patients 80 years or older suggests that age alone should not be a deterrent to anticoagulation in patients aged 80 years or older, consistent with current guideline recommendations [1,2]. In line with this, our study used the CHA_2_DS_2_-VA score, which excludes sex as a variable, for thromboembolic risk stratification. This approach is consistent with the 2024 ESC guideline, which recommends the CHA_2_DS_2_-VA score over CHA_2_DS_2_-VASc due to concerns that female sex functions as a risk modifier only in the presence of other factors [1,28,29]. By removing sex as a determinant, the CHA_2_DS_2_-VA score provides a simplified and more inclusive tool to guide anticoagulation therapy in older adults with AF.

In clinical practice, the choice between standard-dose and low-dose NOACs often relies on the clinician’s subjective assessment rather than strict adherence to guideline-based criteria. The SAKURA AF Registry, which included 3266 patients, revealed that 20–30% of NOAC users were prescribed inappropriately reduced doses despite not meeting established criteria for dose adjustment [30]. This tendency toward underdosing may compromise the efficacy of anticoagulation. Indeed, a meta-analysis of Asian patients from the RE-LY and ENGAGE AF-TIMI 48 trials demonstrated that compared to low-dose NOACs, standard-dose NOACs significantly reduced the risk of ischemic stroke and systemic embolism without a statistically significant difference in major bleeding, ICH, or life-threatening bleeding [27]. These insights emphasize that dose reduction should be limited to patients with clear clinical indications, such as impaired renal function, low body weight, or concomitant medications, and not be based solely on advanced age [31,32]. Our study supports this approach, as clinical outcomes remained favorable, even in very elderly patients treated with DOACs. These findings underscore that advanced age, by itself, should not be a reason to avoid prescribing standard-dose DOACs, provided dosing guidelines are followed. In daily practice, careful attention to renal function, body weight, and concomitant medications remains essential when selecting and adjusting DOAC therapy. Regular monitoring—including periodic reassessment of bleeding risk and renal function—can further support safe and effective anticoagulation in this vulnerable population. Importantly, our study addresses a critical evidence gap by focusing on East Asian patients aged 80 years or older—an underrepresented population in prior randomized trials and real-world registries. Given their heightened vulnerability to both thromboembolic and bleeding events, our findings contribute valuable population-specific data that help inform anticoagulation decisions in this growing subgroup. Taken together, our findings highlight the importance of individualized, evidence-based anticoagulation strategies. For clinicians managing very elderly East Asian patients with AF, our data support not withholding oral anticoagulation on the basis of age alone. When DOACs are prescribed on-label and key parameters—renal function, body weight, and interacting drugs—are periodically reassessed, safety appears acceptable even in those aged ≥80 years.

This study has several limitations. First, it was a retrospective, single-center analysis, which may introduce selection bias, and limit the generalizability of the findings. Second, the sample size of patients receiving warfarin was relatively small, compared to those receiving DOACs, which may have reduced the statistical power to detect differences between treatment groups. This limited sample size also constrains the ability to perform meaningful age-stratified analyses within the warfarin group, and thus caution is warranted when interpreting subgroup comparisons. Third, although multivariable adjustments were performed, residual confounding due to unmeasured variables cannot be ruled out. While methods such as propensity score adjustment or inverse probability weighting could potentially enhance covariate balance between treatment groups, the relatively small sample size—particularly in the warfarin group—limited our ability to apply these approaches without compromising model stability or statistical power. Lastly, bleeding and ischemic events were identified through retrospective medical record review, which may have led to underreporting of minor or asymptomatic events.

## 5. Conclusions

In this retrospective study of elderly East Asian patients with atrial fibrillation, clinical outcomes did not significantly differ between patients aged 70–79 and those aged ≥80 years. Despite numerically higher bleeding rates in the very elderly, DOACs, when appropriately dosed, remained safe and effective. These findings indicate that age alone should not preclude anticoagulation; decisions should prioritize guideline-directed dosing and comorbidity management.

## Figures and Tables

**Table 1:** Baseline characteristics.

	DOAC		Warfarin		*p*-Value	*p*-Value
	Age 70–79 (*n* = 242)	Age ≥ 80 (*n* = 203)	Age 70–79 (*n* = 35)	Age ≥ 80 (*n* = 22)	Age 70–79 vs. Age ≥ 80 (DOAC Group)	Age 70–79 vs. Age ≥ 80 (Warfarin Group)
Age	75.9 ± 2.5	83.4 ± 3.1	75.5 ± 2.6	83.1 ± 2.9	<0.001	<0.001
Sex (male)	120 (49.6)	76 (37.4)	10 (28.6)	6 (27.3)	0.010	0.915
BMI	25.3 ± 9.7	24.1 ± 3.9	24.9 ± 2.9	24.5 ± 4.8	0.102	0.726
Height (cm)	159.4 ± 11.6	156.8 ± 8.8	156.4 ± 6.8	152.6 ± 7.8	0.007	0.059
Weight (kg)	63.1 ± 10.6	59.3 ± 11.5	60.9 ± 7.5	57.3 ± 13.3	<0.001	0.263
Hemoglobin	13.3 ± 1.6	12.6 ± 1.8	12.9 ± 1.9	11.8 ± 1.8	<0.001	0.042
Platelet	214.8 ± 63.5	221.7 ± 70.9	210.0 ± 54.6	190.0 ± 73.2	0.282	0.245
BUN	20.1 ± 32.2	18.6 ± 7.4	17.6 ± 5.7	29.0 ± 35.2	0.515	0.158
Creatinine	0.96 ± 0.5	0.94 ± 0.6	1.0 ± 0.7	1.5 ± 1.7	0.593	0.185
AST	29.3 ± 23.1	26.3 ± 14.5	23.2 ± 8.9	30.4 ± 26.2	0.101	0.148
ALT	22.9 ± 13.9	21 ± 16.9	19.8 ± 9.6	20.9 ± 18.4	0.212	0.771
eGFR (CKD-EPI)	71.7 ± 17.6	67.9 ± 17.5	67.3 ± 22.1	53.2 ± 21.2	0.024	0.020
Alcohol	19 (7.9)	13 (6.4)	1 (2.9)	2 (9.1)	0.556	0.553
Smoking	27 (11.2)	9 (4.4)	1 (2.9)	1 (4.5)	0.010	1.000
Hypertension	157 (64.9)	149 (73.4)	22 (62.9)	11 (50.0)	0.053	0.339
DM	62 (25.6)	53 (25.1)	15 (42.9)	7 (31.8)	0.907	0.405
PTE/DVT	1 (0.4)	3 (1.5)	4 (11.4)	0 (0.0)	0.335	0.151
CVA	30 (12.4)	25 (12.3)	3 (8.6)	5 (22.7)	0.979	0.239
CHF	17 (7.0)	17 (8.4)	3 (8.6)	4 (18.2)	0.593	0.411
CKD	4 (1.7)	8 (3.9)	1 (2.9)	2 (9.1)	0.154	0.553
COPD	8 (3.3)	11 (5.4)	0 (0.0)	3 (13.6)	0.272	0.053
CAD	39 (16.1)	22 (10.8)	5 (14.3)	8 (36.4)	0.107	0.053
CHA2DS2-VA score	3.1 ± 1.3	3.5 ± 1.1	3.2 ± 1.4	3.8 ± 1.6	0.003	0.157
Use of antiplatelet drugs	15 (6.2)	4 (2.0)	1 (2.9)	2 (9)	0.033	0.553

Data are shown as mean ± SD or *n* (%). ALT = alanine aminotransferase; AST = aspartate aminotransferase; BMI = body mass index; BUN = blood urea nitrogen; CAD = coronary artery disease; CHF = congestive heart failure; CKD = chronic kidney disease; COPD = chronic obstructive pulmonary disease; CVA = cerebrovascular accident; DM = diabetes mellitus; DOAC = direct oral anticoagulant; eGFR = estimated glomerular filtration rate; PTE/DVT = pulmonary thromboembolism/deep vein thrombosis.

**Table 2:** Clinical outcomes.

	OAC	Age 70–79	Age ≥ 80	*p*-Value (Event Rate)	Univariate HR (95% CI)	*p*-Value	Multivariate HR ** (95% CI)	*p*-Value
All-cause death	DOAC	25 (10.3)	13 (6.4)	0.173	1.021 (0.519–2.009)	0.953	0.545 (0.265–1.121)	0.099
Ischemic stroke/ systemic thromboembolism		23 (9.5)	13 (6.4)	0.295	1.204 (0.595–2.436)	0.605	0.611 (0.296–1.259)	0.182
Major bleeding		27 (11.2)	21 (10.3)	0.878	1.885 (1.058–3.360)	0.031	0.832 (0.456–1.518)	0.549
ICH		4 (1.7)	5 (2.5)	0.738	2.816 (0.749–10.590)	0.126	1.815 (0.446–7.390)	0.406
All-cause death	Warfarin	6 (17.1)	2 (9.1)	0.466	0.810 (0.161–4.075)	0.798	0.437 (0.071–2.699)	0.373
Ischemic stroke/ systemic thromboembolism		5 (14.3)	4 (18.2)	0.722	2.695 (0.694–10.458)	0.152	0.747 (0.099–5.626)	0.777
Major bleeding		2 (5.7)	5 (22.7)	0.095	6.929 (1.328–36.159)	0.022	3.617 (0.600–21.804)	0.161
ICH		2 (5.7)	0 (0.0)	0.518	0.026 (0.000–59766.431)	0.625	0.964 (0.500–1.858)	0.912

** Adjusted by sex, BMI, eGFR, HTN, DM, CKD, history of CVA, history of CHF, history of COPD, history of CAD, CHA2DS2-VA score, and use of antiplatelet drugs. Data are shown as mean ± SD or *n* (%). CI = confidence interval; DOAC = direct oral anticoagulant; HR = hazard ratio; ICH = intracranial hemorrhage; OAC = oral anticoagulant.

**Table 3:** Sex differences in clinical outcomes within DOAC and warfarin groups.

	OAC	Male (Event Rate)	Female (Event Rate)	Adjusted HR ** (95% CI)	*p*-Value
All-cause death	DOAC	19 (9.7)	19 (7.6)	1.071 (0.527–2.176)	0.849
Ischemic stroke/ systemic thromboembolism		13 (6.6)	23 (9.2)	0.647 (0.301–1.389)	0.264
Major bleeding		20 (10.2)	28 (11.2)	0.944 (0.859–1.038)	0.573
ICH		5 (2.6)	4 (1.6)	1.212 (0.263–5.582)	0.805
All-cause death	Warfarin	1 (6.3)	7 (17.1)	0.815 (0.418–1.588)	0.548
Ischemic stroke/ systemic thromboembolism		3 (18.7)	6 (14.6)	0.191 (0.018–1.991)	0.166
Major bleeding		1 (6.3)	6 (14.6)	0.328 (0.034–3.176)	0.336
ICH		0 (0.0)	2 (4.9)	0.824 (0.423–1.605)	0.570

** Adjusted by age, BMI, eGFR, HTN, DM, CKD, history of CVA, history of CHF, history of COPD, history of CAD, and use of antiplatelet drugs. Data are shown as mean ± SD or *n* (%). CI = confidence interval; DOAC = direct oral anticoagulant; HR = hazard ratio; ICH = intracranial hemorrhage; OAC = oral anticoagulant.

## Data Availability

All data presented in the additional table are available.

## References

[1] Van Gelder I.C., Rienstra M., Bunting K.V., Casado-Arroyo R., Caso V., Crijns H., De Potter T.J.R., Dwight J., Guasti L., Hanke T. (2024). 2024 ESC Guidelines for the management of atrial fibrillation developed in collaboration with the European Association for Cardio-Thoracic Surgery (EACTS). Eur. Heart J..

[2] Joglar J.A., Chung M.K., Armbruster A.L., Benjamin E.J., Chyou J.Y., Cronin E.M., Deswal A., Eckhardt L.L., Goldberger Z.D., Gopinathannair R. (2024). 2023 ACC/AHA/ACCP/HRS Guideline for the Diagnosis and Management of Atrial Fibrillation: A Report of the American College of Cardiology/American Heart Association Joint Committee on Clinical Practice Guidelines. J. Am. Coll. Cardiol..

[3] Wolf P.A., Abbott R.D., Kannel W.B. (1991). Atrial fibrillation as an independent risk factor for stroke: The Framingham Study. Stroke.

[4] Oancea A.F., Jigoranu R.A., Morariu P.C., Miftode R.S., Trandabat B.A., Iov D.E., Cojocaru E., Costache I.I., Baroi L.G., Timofte D.V. (2023). Atrial Fibrillation and Chronic Coronary Ischemia: A Challenging Vicious Circle. Life.

[5] Andreotti F., Geisler T., Collet J.P., Gigante B., Gorog D.A., Halvorsen S., Lip G.Y.H., Morais J., Navarese E.P., Patrono C. (2023). Acute, periprocedural and longterm antithrombotic therapy in older adults: 2022 Update by the ESC Working Group on Thrombosis. Eur. Heart J..

[6] Lip G.Y., Frison L., Halperin J.L., Lane D.A. (2011). Comparative validation of a novel risk score for predicting bleeding risk in anticoagulated patients with atrial fibrillation: The HAS-BLED (Hypertension, Abnormal Renal/Liver Function, Stroke, Bleeding History or Predisposition, Labile INR, Elderly, Drugs/Alcohol Concomitantly) score. J. Am. Coll. Cardiol..

[7] Scharf R.E. (2021). Thrombocytopenia and Hemostatic Changes in Acute and Chronic Liver Disease: Pathophysiology, Clinical and Laboratory Features, and Management. J. Clin. Med..

[8] Dalgaard F., Xu H., Matsouaka R.A., Russo A.M., Curtis A.B., Rasmussen P.V., Ruwald M.H., Fonarow G.C., Lowenstern A., Hansen M.L. (2020). Management of Atrial Fibrillation in Older Patients by Morbidity Burden: Insights From Get With The Guidelines-Atrial Fibrillation. J. Am. Heart Assoc..

[9] Connolly S.J., Ezekowitz M.D., Yusuf S., Eikelboom J., Oldgren J., Parekh A., Pogue J., Reilly P.A., Themeles E., Varrone J. (2009). Dabigatran versus warfarin in patients with atrial fibrillation. N. Engl. J. Med..

[10] Giugliano R.P., Ruff C.T., Braunwald E., Murphy S.A., Wiviott S.D., Halperin J.L., Waldo A.L., Ezekowitz M.D., Weitz J.I., Spinar J. (2013). Edoxaban versus warfarin in patients with atrial fibrillation. N. Engl. J. Med..

[11] Granger C.B., Alexander J.H., McMurray J.J., Lopes R.D., Hylek E.M., Hanna M., Al-Khalidi H.R., Ansell J., Atar D., Avezum A. (2011). Apixaban versus warfarin in patients with atrial fibrillation. N. Engl. J. Med..

[12] Patel M.R., Mahaffey K.W., Garg J., Pan G., Singer D.E., Hacke W., Breithardt G., Halperin J.L., Hankey G.J., Piccini J.P. (2011). Rivaroxaban versus warfarin in nonvalvular atrial fibrillation. N. Engl. J. Med..

[13] Chao T.F., Chiang C.E., Liao J.N., Chen T.J., Lip G.Y.H., Chen S.A. (2020). Comparing the Effectiveness and Safety of Nonvitamin K Antagonist Oral Anticoagulants and Warfarin in Elderly Asian Patients With Atrial Fibrillation: A Nationwide Cohort Study. Chest.

[14] Yamashita Y., Hamatani Y., Esato M., Chun Y.H., Tsuji H., Wada H., Hasegawa K., Abe M., Lip G.Y.H., Akao M. (2016). Clinical Characteristics and Outcomes in Extreme Elderly (Age >/= 85 Years) Japanese Patients With Atrial Fibrillation: The Fushimi AF Registry. Chest.

[15] Bae J.S., Ahn J.H., Tantry U.S., Gurbel P.A., Jeong Y.H. (2018). Should Antithrombotic Treatment Strategies in East Asians Differ from Caucasians?. Curr. Vasc. Pharmacol..

[16] Jeong Y.H. (2014). “East asian paradox”: Challenge for the current antiplatelet strategy of “one-guideline-fits-all races” in acute coronary syndrome. Curr. Cardiol. Rep..

[17] Kim H.K., Tantry U.S., Smith S.C., Jeong M.H., Park S.J., Kim M.H., Lim D.S., Shin E.S., Park D.W., Huo Y. (2021). The East Asian Paradox: An Updated Position Statement on the Challenges to the Current Antithrombotic Strategy in Patients with Cardiovascular Disease. Thromb. Haemost..

[18] Goto S., Zhu J., Liu L., Oh B.H., Wojdyla D.M., Aylward P., Bahit M.C., Gersh B.J., Hanna M., Horowitz J. (2014). Efficacy and safety of apixaban compared with warfarin for stroke prevention in patients with atrial fibrillation from East Asia: A subanalysis of the Apixaban for Reduction in Stroke and Other Thromboembolic Events in Atrial Fibrillation (ARISTOTLE) Trial. Am. Heart J..

[19] Hori M., Matsumoto M., Tanahashi N., Momomura S., Uchiyama S., Goto S., Izumi T., Koretsune Y., Kajikawa M., Kato M. (2012). Rivaroxaban vs. warfarin in Japanese patients with atrial fibrillation-the J-ROCKET AF study. Circ. J..

[20] Yamashita T., Koretsune Y., Yang Y., Chen S.A., Chung N., Shimada Y.J., Kimura T., Miyazaki K., Abe K., Mercuri M. (2016). Edoxaban vs. Warfarin in East Asian Patients With Atrial Fibrillation—An ENGAGE AF-TIMI 48 Subanalysis. Circ. J..

[21] Cha M.J., Choi E.K., Han K.D., Lee S.R., Lim W.H., Oh S., Lip G.Y.H. (2017). Effectiveness and Safety of Non-Vitamin K Antagonist Oral Anticoagulants in Asian Patients With Atrial Fibrillation. Stroke.

[22] Hori M., Connolly S.J., Zhu J., Liu L.S., Lau C.P., Pais P., Xavier D., Kim S.S., Omar R., Dans A.L. (2013). Dabigatran versus warfarin: Effects on ischemic and hemorrhagic strokes and bleeding in Asians and non-Asians with atrial fibrillation. Stroke.

[23] Wong K.S., Hu D.Y., Oomman A., Tan R.S., Patel M.R., Singer D.E., Breithardt G., Mahaffey K.W., Becker R.C., Califf R. (2014). Rivaroxaban for stroke prevention in East Asian patients from the ROCKET AF trial. Stroke.

[24] Kaatz S., Ahmad D., Spyropoulos A.C., Schulman S. (2015). Definition of clinically relevant non-major bleeding in studies of anticoagulants in atrial fibrillation and venous thromboembolic disease in non-surgical patients: Communication from the SSC of the ISTH. J. Thromb. Haemost..

[25] Kohsaka S., Katada J., Saito K., Terayama Y. (2018). Safety and effectiveness of apixaban in comparison to warfarin in patients with nonvalvular atrial fibrillation: A propensity-matched analysis from Japanese administrative claims data. Curr. Med. Res. Opin..

[26] Lee K.H., Park H.W., Lee N., Hyun D.Y., Won J., Oh S.S., Park H.J., Kim Y., Cho J.Y., Kim M.C. (2017). Optimal dose of dabigatran for the prevention of thromboembolism with minimal bleeding risk in Korean patients with atrial fibrillation. Europace.

[27] Wang K.L., Lip G.Y., Lin S.J., Chiang C.E. (2015). Non-Vitamin K Antagonist Oral Anticoagulants for Stroke Prevention in Asian Patients With Nonvalvular Atrial Fibrillation: Meta-Analysis. Stroke.

[28] Mikkelsen A.P., Lindhardsen J., Lip G.Y., Gislason G.H., Torp-Pedersen C., Olesen J.B. (2012). Female sex as a risk factor for stroke in atrial fibrillation: A nationwide cohort study. J. Thromb. Haemost..

[29] Wu V.C., Wu M., Aboyans V., Chang S.H., Chen S.W., Chen M.C., Wang C.L., Hsieh I.C., Chu P.H., Lin Y.S. (2020). Female sex as a risk factor for ischaemic stroke varies with age in patients with atrial fibrillation. Heart.

[30] Okumura Y., Yokoyama K., Matsumoto N., Tachibana E., Kuronuma K., Oiwa K., Matsumoto M., Kojima T., Hanada S., Nomoto K. (2017). Current use of direct oral anticoagulants for atrial fibrillation in Japan: Findings from the SAKURA AF Registry. J. Arrhythm..

[31] Lip G.Y., Lane D.A. (2016). Bleeding risk assessment in atrial fibrillation: Observations on the use and misuse of bleeding risk scores. J. Thromb. Haemost..

[32] Wilke T., Bauer S., Mueller S., Kohlmann T., Bauersachs R. (2017). Patient Preferences for Oral Anticoagulation Therapy in Atrial Fibrillation: A Systematic Literature Review. Patient.

